# Effects of an intervention program for female victims of intimate partner violence on psychological symptoms and perceived social support

**DOI:** 10.3402/ejpt.v5.24797

**Published:** 2014-09-12

**Authors:** Nina B. Hansen, Sara B. Eriksen, Ask Elklit

**Affiliations:** Department of Psychology, National Research Centre for Psychotraumatology, University of Southern Denmark, Odense M, Denmark

**Keywords:** Intimate partner violence, domestic violence, psychological treatment, psychological support, social support, mental health consequences, posttraumatic stress disorder, depression, anxiety

## Abstract

**Background:**

Research has documented severe mental health problems in female victims of intimate partner violence (IPV). Therefore, providing effective treatment is pivotal. Few studies have investigated the effects of intervention programs on reducing the harmful consequences of IPV.

**Objective:**

The present study examined the effects of a specific three-phase intervention program for female victims of IPV on psychological symptoms (PTSD, anxiety, and depression) and perceived social support. Given that many of the women dropped out before and during the intervention program, potential differences in initial levels of psychological symptoms, perceived social support, as well as descriptive variables were explored between the women who completed the whole program and the groups of women who dropped out prematurely.

**Method:**

The initial sample consisted of 212 female victims of IPV. Symptoms of PTSD, depression, anxiety, and level of perceived social support were measured with validated scales before the start of the intervention and after completion of each treatment phase.

**Results:**

Results showed a significant effect of the intervention program on reducing psychological symptoms and increasing levels of perceived social support. Effect sizes ranged from medium to very high. Significant positive effects were found for each of the treatment phases. There were no significant differences between the women who completed the whole program and those women who dropped out prematurely in terms of initial level of symptoms and perceived social support as well as descriptive characteristics.

**Conclusions:**

Specifically developed intervention programs for female victims of IPV are effective in reducing the harmful personal consequences of IPV. Future studies should consider employing controlled study designs and address the issue of high drop out rates found in intervention studies.

Intimate partner violence (IPV) is a global problem of tremendous proportions. The World Health Organization (WHO) estimates that 10–50% of women experience physical or sexual abuse by their partner at some point in their lives. Estimations show that 28,000 Danish women between the ages of 16 and 64 years are exposed to IPV during the course of a year (Helweg-Larsen & Frederiksen, 2007). When other forms of abuse, such as psychological violence, are added into the equation, the number of victims increases markedly (Ramsay et al., 2009).

Exposure to IPV can have serious consequences on the victim's physical and mental health (Díez et al., 2009). IPV is associated with various mental health problems in female victims. For example, prevalence rates of posttraumatic stress disorder (PTSD) in female victims of IPV are reported to vary between 24 and 84% (Coker, Weston, Creson, Justice, & Blakeney, 2012; Golding, 1999; Lilly & Graham-Bermann, 2010). Studies also report elevated levels of depression in female victims of IPV. In a review of 18 studies that assessed clinical and non-clinical samples, Golding (1999) found that the average rate of depression among female victims of IPV was 47.6%. Studies have found similar patterns of anxiety in female victims of IPV (Hurwitz, Gupta, Liu, Silverman, & Raj, 2006; Mitchell et al., 2006).

In addition to the various mental health problems associated with IPV, female victims of IPV report having smaller and less supportive social networks than non-abused women (Katerndahl, Burge, Ferrer, Becho, & Wood, 2013). Within trauma research, social support typically represents either the *actual social support* received by the victim or *perceived social support*, that is, the victim's conviction that support is available when needed (Norris & Kaniasty, 1996). Absence of actual social support and lower levels of perceived social support have been identified as risk factors for the development of PTSD in trauma-exposed individuals (Brewin, Andrews, & Valentine, 2000; Ozer, Best, Lipsey, & Weiss, 2003). On the contrary, adequate actual- and perceived support can act as a buffer against the development of PTSD in trauma-exposed victims (Ozer et al., 2003; Robinaugh et al., 2011).

Aside from the extensive individual costs associated with IPV, the problems faced by female victims of IPV also weigh heavily on yearly governmental resources (Helweg-Larsen, Krise, Sørensen, & Brænnum-Hansen, 2010) in terms of costs associated with temporary placements in women shelters, visits to the hospital/the general practitioner, legal processes, and sick days. Providing effective treatment for female victims of IPV is therefore pivotal if we are to reduce both the harmful individual and governmental costs associated with the IPV. A number of intervention programs have been developed to treat trauma-related reactions in victims of various types of trauma (Courtois & Ford, 2009). However, few studies have investigated the effects of interventions programs on reducing the harmful consequences of IPV. Some studies have investigated mainly individual therapy consisting of elements such as empowerment (Johnson & Zlotnick, 2006; Johnson, Zlotnick, & Perez, 2011; Perez, Johnson, & Wright, 2012), self-advocacy (Ford-Gilboe, Wuest, Varcoe, & Merrit-Gray, 2006; Tawari et al., 2010), and cognitive behavioral therapy (CBT; Kubany, Hill, & Owens, 2003; Kubany et al., 2004). Other studies have assessed therapeutic methods such as interpersonal therapy (Zlotnick, Capezza, & Parker, 2010) and eye movement desensitization and reprocessing (EMDR; Colosetti & Thyer, 2000). Most of the aforementioned treatment studies have focused on a few measures of PTSD and depression/anxiety and have overlooked the important association between perceived social support and mental health in victims of IPV (Kamimura, Parekh, & Olsen, 2013). The few studies that have investigated the effects of intervention programs for victims of IPV on reducing depressive symptoms and symptoms of PTSD generally report high effects sizes (Johnson & Zlotnick, 2006; Johnson et al., 2011; Kubany et al., 2004; Zlotnick et al., 2010). Thus, there is evidence to suggest that treatment program target specifically toward female victims of IPV may be effective in reducing the negative outcomes associated with IPV.

The intervention program of interest in the present study was developed by a private Danish organization called “The Mothers’ Aid” (MA). The program initially included 212 female victims of IPV (and their children) who had left their violent partner. The program was based on the work of Mendelsohn et al. (2011) who developed a group intervention program for trauma-exposed adults called *The Trauma Recovery Group* (TRG). We know of only a couple of studies that have investigated the effect of the TRG method on reducing psychological symptoms. In two quantitative studies, Mendelsohn et al. (2011) found that trauma-exposed individuals reported significant reductions in PTSD-, depression-, and dissociative symptomatology during the course of treatment. However, there is a need for further research in this area.

The intervention program in the present study consisted of three separate phases: a stabilization-, a treatment-, and a follow-up phase. The first aim of the study was to examine the effects of the overall intervention program *and* its three individual phases on psychological symptoms (PTSD, depression, and anxiety) and perceived social support among female victims of IPV. Given that many of the women dropped out of the intervention program before (*n*=75) and during the treatment (*n*=67) course, the second aim of this study was to examine possible differences in the initial level of psychological symptoms, perceived social support, and descriptive variables (including demographic characteristics and exposure to violence) between those who dropped out prematurely and those who completed the whole intervention program.

## Methods

### Sample

The initial sample consisted of 212 female victims of IPV. Seventy-five women dropped out of the program before it started and another 67 women dropped out during the course of the treatment. All included women were in contact with the organization MA that helps vulnerable women and their children. They were referred to a special MA intervention program called “Out of the Shadows of Violence” (OSV), which was aimed at helping female victims of IPV and their children. The women were recruited for the program through the general support initiative of MA as well as information brochures distributed to crisis centers for women, emergency departments, and public support centers.

IPV was defined in the present study as any abuse of power by one partner against the other partner in an intimate relationship. It referred to events where one partner dominated, controlled, violated, or victimized the other partner physically, emotionally, and/or sexually. In order to be considered eligible for the intervention program, the women had to have left their violent partner, expressed a desire to move on with their lives following the violent relationship, and have one or more children in the age group of 0–12 years and be able to speak Danish. Furthermore, they were not allowed to participate in any other treatment program throughout the duration of the project.

### The intervention program

The intervention program in the present study was based on the TRG method and the traumatic treatment approach presented by Mendelsohn et al. (2011) and Herman (1997), respectively. However, the TRG manual was not followed completely in this study. For example, the present study sample consisted of female victims of IPV and not victims of trauma in general. This alteration represents an important feature of the present study given that most of the female participants were forced to collaborate with their abusive partner (e.g., because of shared parental responsibilities). Consistent with the TRG method, though, was that the intervention program in this study followed a three-phase stepwise treatment approach.

#### Phase 1: the stabilization program

The overall aim of this stabilization program was to provide the woman with a sense of control over her physical safety as well as her psychological and social situation. More specifically, this program aimed to stabilize the woman's psychological symptoms as well as her financial and economic situation and to provide her and her child/children with physical safety and legal aid. The length of the stabilization program varied from 1 to 12 individual sessions depending on the individual needs of the woman. All sessions were carried out by social workers.

The stabilization program was created due to the fact that many of the women suffered from severe psychological symptoms that resulted in them being unable to profit from standard psychological intervention programs that focus on the processing of traumatic experiences and subsequent reactions. For example, many of the women engaged in self-harming behavior and many women were unable to protect themselves against their former partner's ongoing physical and psychological violence.

An example of specific components of the stabilization program was psychoeducation about psychological reactions to trauma and the rebuilding of everyday routines. In cases where the women were still being exposed to abuse from their former partner, the focus of the stabilization program was to secure them a new place of residence and to help design a potential escape plan, if necessary. With regards to social problems, example of specific actions in the stabilization program was to help the women get to a new residence, to acquire control over her financial situation, and to gain custody of their child/children. Another aspect of the stabilization program was to determine whether the women needed individual- or group treatment.

#### Phase 2: the treatment program

Upon completing the stabilization program, the women went on to participate in the treatment program, which consisted of either individual- or group therapy. Individual sessions (with a psychologist) were offered to those women who did not want to or who were deemed unsuitable to participate in group sessions. The aims of the individual sessions were to help the women process traumatic experiences, to reduce their psychological symptoms, to provide them with a better understanding of their child's (children) situation, and to enhance their ability to talk with the child (children) about the violence. The length of the individual treatment varied from 1 to 12 sessions depending on the needs of the woman. Sessions were generally scheduled to every 14th day, but were scheduled more frequently in special circumstances. Each session lasted between 1 and 1½ hours.

Group sessions were offered to women who wanted and were deemed suitable to participate in group treatment. The aims of the group sessions were to help the women to process traumatic experiences, to reduce psychological symptoms as well as feelings of guilt, shame, loneliness, and alienation. The group treatment consisted of 14 3-hour sessions scheduled once a week. The sessions were carried out by a psychologist and a social worker. The sessions were clearly structured; they had defined purposes and specific working tasks. All group members were obliged to actively participate in each session. The psychologist and social worker were responsible for ensuring that every group member had their own time to speak. They were also responsible for promoting the exchange of experiences and reducing potential conflict among group members. Each session started with a summary of what happened in the previous session. Thereafter followed the specific topic of the day, and, at the end of each session, the group members were asked to write down what they had learned. The sessions consisted of the following topics: introduction (session 1); the violent experiences (sessions 1–4); mental and physical consequences of the violence (session 5–9); the children's situation (sessions 10–12); the future (session 13); termination (session 14).

#### Phase 3: the follow-up program

The follow-up program aimed to maintain the positive changes displayed by the women and to support them in their new lives without their abusive partner. The follow-up program consisted of 2–4 individual sessions for the women who had completed individual treatment and three group sessions for the women who had completed group treatment. The follow-up sessions took place during the first 6 months after the treatment had been completed. The follow-up sessions focused on the psychological well-being of the women as well as their relationships with their children and former abusive partners.

### Data collection

Data on PTSD severity, depressive and anxiety symptoms, and perceived social support were gathered from all the women during the OSV project period. This data collection represented part of an effort to evaluate the effectiveness of the overall intervention program and its individual intervention phases. Psychological data was gathered at four different time points: T1) before entering the stabilization program, T2) after completion of the stabilization program, T3) after completion of the treatment program, and T4) after completion of the follow-up program. Overall, 212 women were included in project OSV (T1) but 75 women dropped out of the project before entering the stabilization program. Consequently, 137 women completed the stabilization program (T2), 87 women completed both the stabilization and the treatment programs, (T3) and 70 women completed the whole intervention program (T4).

The study was carried out in accordance with the ethical principles of the Declaration of Helsinki and all data were treated anonymously.

### Measures

PTSD severity was assessed at all four time points using the Danish version of the Harvard Trauma Questionnaire (HTQ; Mollica et al., 1992). The HTQ measures the intensity of the three core symptom groups of PTSD (intrusion, avoidance, and arousal) and consists of 30 items, 16 of which correspond to the *DSM-IV*. The answers are scored on a 4-point Likert-type scale (1=not at all, to 4=all the time). The Danish version of the HTQ has shown good reliability and validity (Bach, 2003). In the present study, the Cronbach's alpha value for the total HTQ scale at the start of the treatment was 0.87.

Symptoms of anxiety and depression were assessed at all four time points using the Danish version of the Hopkins Symptoms Checklist (HSCL; Derogatis, Lipman, Rickels, Uhlenhuth, & Covi, 1974). The HSCL consists of 25 items. The first 10 items measure symptoms of anxiety and the last 15 items measure symptoms of depression. Responses are scored on a 4-point Likert-type scale (1=not at all, to 4=very much). The HSCL has shown good reliability and validity (Nettelbladt, Hansson, Stefansson, Borgquist, & Nordström, 1993); however, it has not yet been validated in a Danish sample. In the present study, the Cronbach's alpha value at treatment start was 0.76 for the anxiety subscale and 0.88 for the depression subscale.

Perceived social support was assessed at all four time points using the Danish version of the Crisis Support Scale (CSS; Joseph, Andrews, Williams, & Yule, 1992). The scale comprises the following seven items: 1) perceived availability of someone to listen; 2) contact with people in a similar situation; 3) the ability to express oneself; 4) received sympathy and support; 5) practical support; 6) the experience of being let down; and 7) general satisfaction with social support. The total seven-item scale is usually referred to as perceived social support. Responses are rated on a 7-point Likert-type scale (1=never, to 7=always). The Danish version of the CSS has shown good reliability and validity (Elklit, Pedersen, & Jind, 2001). In the present study, the Cronbach's alpha value for the total CSS scale measured at treatment start was 0.78.

### Statistical analyses

Paired sample *t*-tests were conducted to assess the effect of the overall intervention program on PTSD severity, depressive symptoms, anxiety symptoms, and perceived social support among the women who completed the whole program. In addition, paired sample *t*-tests were conducted to assess the effect of the individual intervention phases (the stabilization-, treatment-, and follow-up programs) on psychological symptoms and perceived social support. Effect sizes were calculated using Cohen's d.

In order to explore potential differences between the different attrition groups and the group completing the whole intervention, the study sample was divided into four groups. Group 1 consisted of women who dropped out of the intervention program before it started. Group 2 consisted of women who only completed the stabilization program. Group 3 consisted of women who completed the stabilization- and treatment programs. Group 4 consisted of women who completed the whole intervention program. A one-way between-groups analysis of variance was conducted to explore associations between baseline psychological symptoms, perceived social support, and level of treatment participation (groups 1–4). Due to groups having unequal sample sizes, Tukey HSD post-hoc comparisons were conducted to explore between group variations. Chi-square tests for independence were used to explore potential relationship between the descriptive variables represented in Table 1 and levels of treatment participation (groups 1–4). All analyses were conducted using the Statistical Package for Social Sciences (SPSS) version 21 and statistical significance was set at p≤0.05

**Table 1:** Descriptive characteristics and psychological symptoms before the start of the intervention on the women completing the whole intervention program and groups of women dropping out prematurely

	Women completing the whole intervention program (*n*=70)	Women completing the stabilization program and treatment program (*n*=17)	Women completing only the stabilization program (*n*=50)	Women dropping out before the start of the treatment (*n*=75)
	
	*n* (%)	*n* (%)	*n* (%)	*n* (%)
Civil Status				
Single	42 (76.4)	10 (71.4)	37 (75.5)	60 (89.6)
Married	6 (10.9)	2 (14.3)	4 (8.2)	5 (7.5)
Separated	2 (3.6)	2 (14.3)	5 (10.2)	2 (2.9)
With a partner	5 (9.1)	–	3 (6.1)	–
Years of education				
>10 years	17 (32.1)	6 (46.2)	22 (50)	23 (34.8)
<10 year	36 (67.9)	7 (53.8)	22 (50)	43 (65.2)
Work status				
Ordinary work	32 (59.3)	7 (50)	23 (47.9)	24 (36.4)
Under education	6 (11.1)	3 (21.4)	7 (14.6)	12 (18.2)
Unemployed	16 (29.6)	4 (28.6)	16 (33.3)	23 (34.8)
Public support	–	–	2 (4.2)	7 (10.6)
Violence frequency				
Daily	15 (34.9)	4 (28.6)	10 (26.3)	13 (28.3)
Weekly	14 (32.5)	8 (57.1)	18 (47.4)	21 (45.7)
Monthly	14 (32.5)	2 (14.3)	9 (23.7)	10 (21.7)
One time	–	–	–	1 (2.2)
Not sure	–	–	1 (2.6)	1 (2.2)
Violence duration				
<1 year	1 (2.3)	–	1 (2.6)	3 (5.6)
1–4 years	18 (41.9)	7 (50)	23 (60.5)	30 (55.6)
>5 years	24 (55.8)	7 (50)	14 (36.8)	21 (38.8)
Psychological symptoms before interventions start	M (SD)	M (SD)	M (SD)	M (SD)
PTSD severity	46.71 (9.15)	45.78 (10.93)	43.02 (8.61)	45.88 (10.28)
Depressive symptoms	35.28 (8.48)	35.00 (8.81)	36.74 (8.32)	35.50 (9.25)
Anxiety symptoms	27.74 (5.31)	27.73 (5.33)	26.76 (5.22)	27.08 (5.29)
Perceived social support before intervention start	27.83 (6.86)	26.60 (6.11)	26.96 (7.96)	26.11 (7.64)

## Results

### Descriptive characteristics

Descriptive characteristics and psychological symptoms before start of treatment of the whole study sample and the different treatment participation groups are presented in Table 1. Unfortunately, a number of women did not provide descriptive information and information about the age of the woman and exact number of children was not available.

### Intervention effect on psychological symptoms and perceived social support

Paired samples *t*-tests conducted on the data for the 70 women completing the whole treatment program revealed significant reductions in all psychological symptoms following the intervention program. Effects sizes were in the very high range, the highest being for anxiety symptoms. Paired samples *t*-test also revealed a significant increase in levels of perceived social support following the intervention program. The effect size was in the high range. Means (M), standard deviations (SD), *t*-values, and effect sizes are presented in Table 2.

**Table 2:** Intervention effect on psychological symptoms and perceived social support

	Before intervention M (SD)	After intervention M (SD)	*t*-test	Effect size (d)
PTSD severity	46.71 (9.15)	35.15 (10.23)	*t*(56)=6.50*	1.19
Depressive symptoms	35.39 (8.61)	23.20 (6.55)	*t*(52)=9.1*	1.59
Anxiety symptoms	27.62 (5.38)	17.03 (4.35)	*t*(50)=12.50*	2.16
Perceived social support	27.72 (6.85)	32.79 (6.85)	*t*(53)=−5.77*	0.88

* *p*<0.01.

### Changes in psychological symptoms and perceived social support following each intervention phase

#### The stabilization program

Paired sample *t*-tests conducted on data for the women who completed the stabilization program revealed significant reductions in PTSD severity (*t*(103)=7.34; *p*<0.01, *d=0*.54), depressive symptoms (*t*(104)=6.29; *p*<0.01; *d*=0.56), and anxiety symptoms (*t*(109)=6.30; *p*<0.01; *d=0*.47) from before to after completion of the stabilization program. In addition, there was a significant increase in levels of perceived social support (*t*(113)=−9.18; *p*<0.01; *d*=0.82) from before to after completion of the stabilization program.

#### The treatment program

Paired sample *t*-tests conducted on the data for the women who completed the treatment program revealed significant reductions in PTSD severity (*t*(78)=3.09; *p*<0.01; *d=0*.24), depressive symptoms (*t*(77)=5.16; *p*<0.01*, d=0*.56), and anxiety symptoms (*t*(76)=8.31; *p*<0.01; *d=*1.15) from before to after completion of the treatment program. There was no significant difference in levels of perceived social support.

#### The follow-up program

Paired sample *t*-tests conducted on data for all women who completed the follow-up program revealed significant reductions in PTSD severity (*t*(68)=2.31; *p*<0.05; *d=0*.26) and depressive symptoms (*t*(63)=2.93; *p*<0.01; *d=0*.37) from before to after completion of the follow-up program. There were no significant differences in anxiety symptoms and levels of perceived social support.

**Fig. 1:**
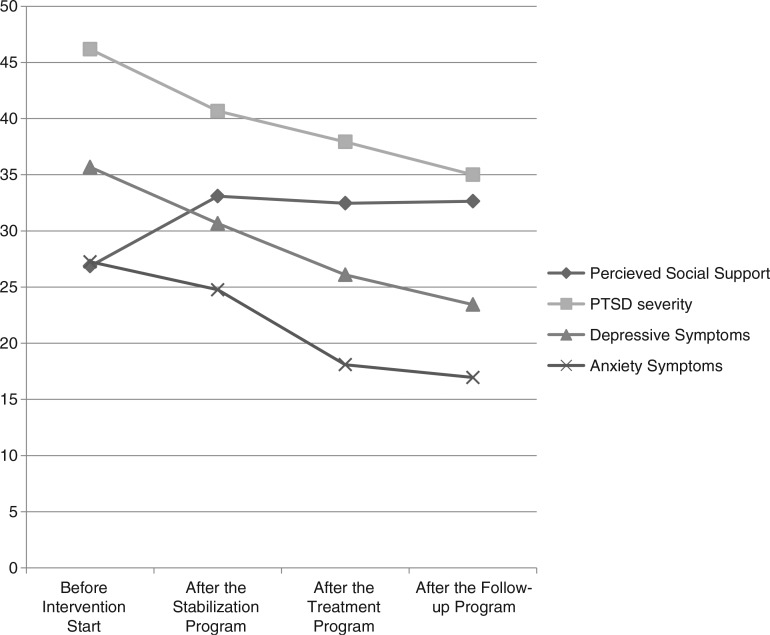
The development of PTSD severity, depressive symptoms, anxiety symptoms, and perceived social support over the individual treatment phases.

The development of psychological symptoms and levels of perceived social support throughout the individual intervention phases are illustrated in Fig. 1.

### Differences in psychological symptoms, perceived social support and descriptive variables according to level of participation

One-way ANOVA revealed no significant associations between levels of treatment participation and levels of psychological symptoms and perceived social support measured before the start of the intervention. Chi-square test of independence also indicated no significant associations between levels of participation and civil status, years of education, work status, violence frequency, and duration of violence exposure.

## Discussion

The results of this study showed significant effects of a specific three-phased intervention program (OSV) in reducing psychological symptoms and in increasing levels of perceived social support among women formerly exposed to IPV. These results support findings from previous studies that report significant reductions in PTSD, depression, and anxiety in female victims of IPV following participation in intervention programs (Colosetti & Thyer, 2000; Graham-Bermann & Miller, 2013; Johnson et al., 2011; Kubany et al., 2004; Mendelsohn et al., 2011). The effect sizes in the present study were very high (*d=*0.88–2.16), and are similar to effect sizes in other treatment studies with female victims of IPV (Graham-Bermann & Miller, 2013; Johnson & Zlotnick, 2006). When taking into account the specific psychological problems, the intervention program in the present study demonstrated the highest effect on the women's levels of anxiety (*d*=2.16) and depression (*d*=1.59). However, the intervention program also had a high effect on symptoms of PTSD (*d*=1.19). These findings highlight the importance of not only focusing on PTSD symptomatology but also on levels of anxiety and depression when treating and investigating the harmful effects of IPV. One possible explanation for the very high effect sizes found in relation to anxiety and depression could be the frequency and duration of the IPV reported in this sample of women. In the present study, 95.4% of the women had lived with their violent partner for more than 1 year, and 75.3% of them had been exposed to violence on a daily or weekly basis. It is possible that PTSD symptomatology found in this sample of women was persistent rather than acute due to a prolonged period of trauma exposures, which is similar to situations known to trigger complex PTSD (Herman, 1992).

A unique feature of the present study was its focus on perceived social support. Research has shown that actual and perceived social support can act as a buffer against the harmful effects of traumatic experiences (Escribá-Agüir et al., 2010; Ozer et al., 2003). Thus, increasing social support may be an important therapeutic target. The women in this study reported an increase in levels of perceived social support during the intervention program; however, this increase was only statistically significant in relation to the first phase of the program (the stabilization program). Johnson et al. (2011) found similar results in their CBT treatment study where participants experienced a significant increase in levels of social support after treatment; however, this effect was only present at the 1-week- and not the 6-month follow-up measure. Johnson et al. (2011) suggest that an increase in social support can be interpreted as a side gain of the women's emerging sense of well-being rather than a specific goal for the treatment of IPV victims. Furthermore, it is possible that merely entering the OSV program had a significant effect on levels of perceived social support in this present sample and that this positive effect was maintained during the treatment, although it did not increase any further. Even though this study found a positive increase in the women's levels of perceived social support, the majority of the women in the present sample were living and taking care of their children on their own (85.5%), which might limit the amount of support provided to them on a daily basis. It is possible that the lack of continuing positive changes in perceived social support during the course of treatment merely reflects the women's realistic judgment of the amount of support available to them.

The present study also examined the effects of the individual treatment phases (the stabilization-, treatment-, and follow-up program) of the intervention program on psychological symptoms and perceived social support. Aside from symptoms of anxiety, which did not reduce significantly after completion of the follow-up phase, the results revealed significant reduction in all other psychological symptoms following completion of each of the individual treatment phases. Levels of perceived social support only increased significantly following the completion of the stabilization program. With regards to the stabilization program, the effect sizes ranged between medium for PTSD severity (*d=*0.54) and high for perceived social support (*d*=0.82). The stabilization program focused particularly on using ego-supportive techniques as opposed to the symptom specific treatment used in the treatment program, which may explain why a higher effect size was found for perceived social support than psychological symptoms. With regards to the treatment program, effect sizes ranged between low for PTSD (*d=*0.24) and high for anxiety (*d=*1.15) with depression situated in the medium range. With regards to the follow-up phase, the women reported further but smaller reductions in symptoms of PTSD (*d=*0.26) and depression (*d*=0.37). When comparing effect sizes across the three intervention phases, the lowest effect sizes were found for the follow-up phase. Even though the women received considerably less sessions in this part of the intervention program compared to the other phases, they still showed improvement on most of the outcomes measures. However, it is difficult to determine whether these improvements were due to the follow-up sessions or the result from the continuing effects of the earlier intervention phases. Furthermore, it is possible that time itself may have caused these positive outcomes given that this study had no control group to compare the results with.

Many women dropped out of the intervention program both before and during the intervention program. Therefore, the present study explored whether there were any differences in the initial levels of psychological symptoms and perceived social support as well as descriptive characteristics between the women who completed the whole program and those who dropped out early. No statistically significant differences were found in the present study. Unfortunately, information on important variables such as age and the women's number of children were lacking in the present study. There appears to be a general problem with retaining victims of IPV in intervention programs given that other studies also report high drop-outs rates (Johnson et al., 2011; Kubany et al., 2004). This is an important problem that needs to be addressed in future intervention studies.

### Limitations and implications

The results of the present study have to be interpreted in light of some limitations. Given that this study did not include a control group, it is difficult to determine whether the effects of the OSV program were due to the intervention itself or other factors such as the passing of time. However, assigning victims of IPV to a wait-list control group and denying them treatment poses serious ethical challenges, which must be taken into consideration when designing future intervention studies. A possible solution could be to use a “treatment as usual group” as a control group. This was unfortunately not an option in this study given that there are no standardized treatments for victims of IPV in Denmark at present.

Another implication for future studies is that positive intervention effects have been found across different intervention methods. The treatment manual of the OSV program was founded in the theoretical and methodological reflections of Mendelsohn et al. (2011) whereas other intervention programs are based on other designs (e.g., CBT, interpersonal therapy, and advocacy). In order to determine which intervention programs are most successful in helping female victims of IPV, research that compares the effects of different treatment manuals is needed. In addition, there is a need for more research that explores potential and possible different effects of individual versus group therapy for female victims of IPV. The OSV program had a special design in which the participants received the treatment either individually or in groups. The distribution of individual versus group treatment was determined by different factors such as the women's stability, ability to function in a group successfully, and personal preferences. This choice of design posed a logistic challenge because it was more difficult to organize both types of treatments than only one treatment. However, this type of intervention design was expected to be more sensitive to the women's individual needs. To fully validate this argument, it has to be determined whether there were differences between the women who received individual treatment and those that received group treatment. However, it was not possible to explore potential differences between these two groups because data for these two groups had not been separated in the data collection of the present study. In addition, it is not known whether the included women had a higher preference for group therapy or individual therapy. These are important research questions that need to be addressed in future studies.

Another limitation concerns the overall dropout rate of 33% of the participants in the present study, which limits the generalizability of the results. Thus, the results may not be representative for all of the women who were initially included in the program. However, no differences in initial levels of psychological symptoms and perceived social support as well as descriptive variables were identified between the groups of women with different dropout rates. This indicates that the final study sample was similar to the initially included sample in relation to these variables. Other intervention studies report similar or even higher dropout rates (Johnson et al., 2011; Kubany et al., 2004). Therefore, problems with retaining victims of IPV in intervention programs need to be addressed in future intervention programs.

## Conclusion

Specific intervention programs for female victims of IPV have shown to be effective in treating the harmful personal consequences following this trauma exposure. The current OSV program was found to significantly reduce the women's psychological symptoms across the intervention period. Furthermore, certain parts of the intervention program had a significant positive effect on the women's levels of perceived social support. The division of the intervention program into different phases proved successful given that positive effects were found for each treatment component in relation to the women's psychological symptoms. Future research needs to focus on investigating which types of intervention programs are most successful in reducing the harmful effects of IPV as well as the question on how to reduce dropout rates in intervention programs.
